# Assessment of Phenolic Compounds and Anti-Inflammatory Activity of Ethyl Acetate Phase of *Anacardium occidentale* L. Bark

**DOI:** 10.3390/molecules21081087

**Published:** 2016-08-19

**Authors:** Marina Suênia de Araújo Vilar, Graziene Lopes de Souza, Daniela de Araújo Vilar, Jacqueline Alves Leite, Fernanda Nervo Raffin, José Maria Barbosa-Filho, Fernando Henrique Andrade Nogueira, Sandra Rodrigues-Mascarenhas, Túlio Flávio Accioly de Lima Moura

**Affiliations:** 1 Biotechnology Center, Universidade Federal da Paraíba, João Pessoa-PB 58051-900, Brazil; peritaquimica@yahoo.com.br (M.S.d.A.V.); dani_1011@yahoo.com.br (D.d.A.V.); jacqalvesleite@hotmail.com (J.A.L.); sandra@cbiotec.ufpb.br (S.R.-M.); 2 Department of Pharmacy, Federal University of Rio Grande do Norte, Natal-RN 59012-570, Brazil; graziene.lopes@gmail.com (G.L.d.S.); feraffin@ufrnet.br (F.N.R.); fhanogueira@gmail.com (F.H.A.N.); mouratf@hotmail.com (T.F.A.d.L.M.)

**Keywords:** *Anacardium occidentale*, high-performance liquid chromatography, acute inflammation, phenolic compounds, anti-inflammatory activity

## Abstract

The bark of *A. occidentale* L. is rich in tannins. Studies have described various biological activities of the plant, including antimicrobial, antioxidant, antiulcerogenic and antiinflammatory actions. The objective of this study was to assess the activity of the ethyl acetate phase (EtOAc) of *A. occidentale* on acute inflammation and to identify and quantify its phenolic compounds by HPLC. The method was validated and shown to be linear, precise and accurate for catechin, epicatechin, epigallocatechin and gallic acid. Swiss albino mice (*Mus musculus*) were treated with saline, Carrageenan (2.5%), Indomethacin (10 mg/kg), Bradykinin (6 nmol) and Prostaglandine E2 (5 µg) at different concentrations of EtOAc - *A. occidentale* (12.5; 25; 50; and 100 mg/kg/weight p.o.) for the paw edema test. Challenge was performed with carrageenan (500 µg/mL i.p.) for the doses 50 and 100 mg/kg of EtOAc. Levels of cytokines IL-1, TNF-α, IL-6 and IL-10 were also measured. All EtOAc - *A. occidentale* concentrations reduced the edema. At 50 and 100 mg/kg, an anti-inflammatory response of the EtOAc was observed. Carrageenan stimulus produced a neutrophil count of 28.6% while 50 and 100 mg/kg of the phase reduced this to 14.5% and 9.1%, respectively. The EtOAc extract reduced levels of IL-1 and TNF-α. These results suggest that the EtOAc plays a modulatory role in the inflammatory response. The chromatographic method can be used for the analysis of the phenolic compounds of the EtOAc phase.

## 1. Introduction

*Anacardium occidentale* L. is a medicinal plant native to Brazil known as acajaiba, caju, caju-anão or cajueiro [1]. It is used as an analgesic, diuretic, mouthwash, and for asthenia treatment, respiratory problems, influenza, bronchitis, cough, scurvy, infantile eczema, genital infections, scabies, skin diseases, warts and sores [2]. *A. occidentale* is one of the 71 plant species in the National List of Medicinal Plants of Interest to the Unified Health System (RENISUS), established by the Brazilian Government in 2009 [3].

Phytochemical studies on *A. occidentale* species have shown the presence of eleven classes of different secondary metabolites, although tannins are primarily responsible for the pharmacological actions of the plant. Effective anti-inflammatory activity has also been attributed to these tannins through popular use and based on data from the literature [4,5].

HPLC was the method of choice given its versatility and precision for the analysis of phenolic compounds [6]. HPLC is a useful technique for the separation, identification and quantification of chemical species, and is widely used in pharmaceutical industry laboratories.

Pre-clinical trials conducted using metabolites isolated from *A. occidentale* bark have reported various biological activities of the plant including hypoglycemic [7,8], antimicrobial [9], antioxidant [10], antiulcerogenic [11], anti-ophidian [12] and antileishmanial [13] actions, acetylcholinesterase enzyme inhibition [14] and also an anti-inflammatory effect [4,15]. These studies reveal promising effects of *A. occidentale* in modulating the inflammatory response. These include both anti-edematogenic and antinociceptive effects in addition to action on septic shock. The development of methods for the quality control of pharmaceutical products based on this species is therefore of great importance.

The primary aim of this study was to develop a HPLC method for the analysis of the EtOAc phase of the stem bark of *A. occidentale.* A secondary objective was to seek further evidence of the plant´s anti-inflammatory activity, given the scant knowledge on its influence in modulating the cellular effect during acute inflammation: cellular migration, identification of the cell population involved, and influence of proinflammatory and inflammatory cytokines.

This information can help elucidate the molecular signalling pathways involved in the anti-inflammatory effects of the plant and correlate these with the phytochemicals identified in the EtOAc phase, allowing subsequent development of a more effective drug with proven quality and safety.

## 2. Results and Discussion

### 2.1. Method Development

The gradient elution tested in the development of the HPLC chromatographic method for analysis of the EtOAc phase of *A. occidentale* is shown in Table 1 The first run consisted of an exploratory gradient and resulted in a chromatogram with peaks predominantly in the beginning of the elution, suggesting that most of the components of the phase were polar.

**Figure 1:**
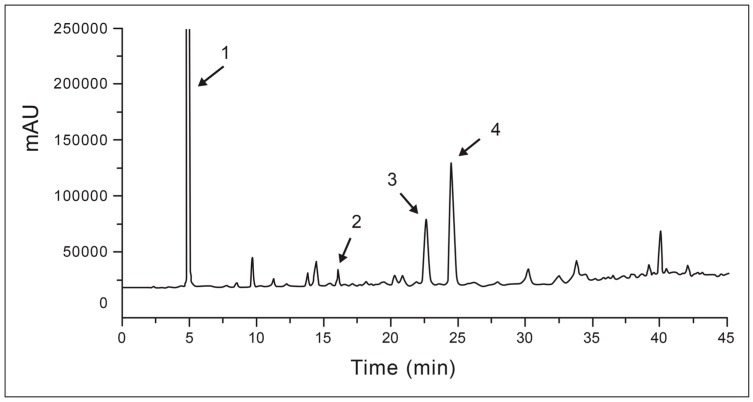
Chromatogram obtained by analyzing EtOAc phase of acetone extract of *A. occidentale* barks under optimized conditions of the analytical method. gallic acid (1); catechin (2); epicatechin (3); and epigallocatechin (4).

Thus, adjustments were made in subsequent runs and the optimal separation and resolution for the peaks and the shortest run time were obtained by using the gradient of run No 7. The chromatogram obtained using the optimized conditions of the method is shown in Figure 1.

Comparison of retention times (RT) and UV absorption spectra of the standards of phenolic compounds and the peaks of the chromatogram obtained for the *A. occidentale* EtOAc phase alone shows that the peaks labelled 1, 2, 3 and 4 in Figure 1 correspond to gallic acid, catechin, epicatechin and epigallocatechin, respectively.

**Figure 2:**
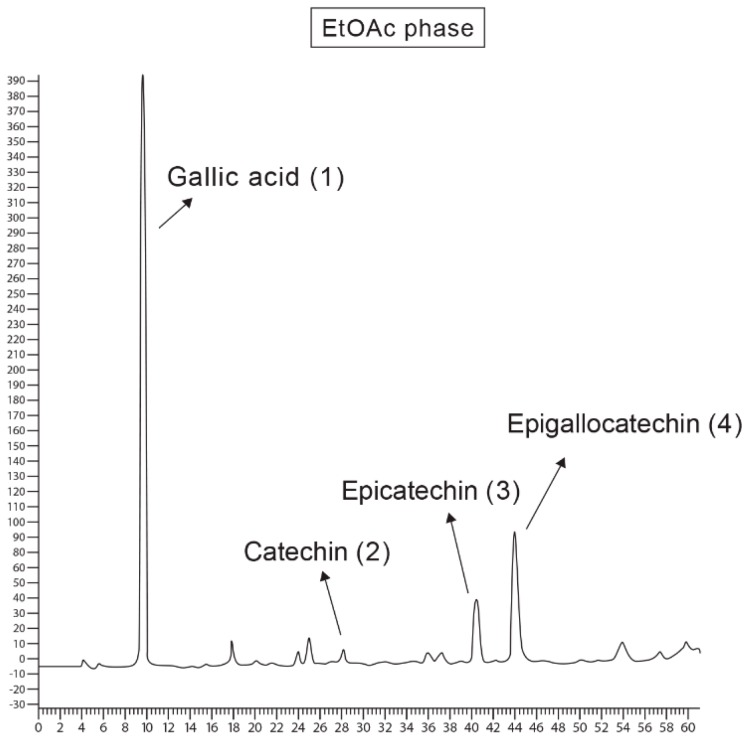

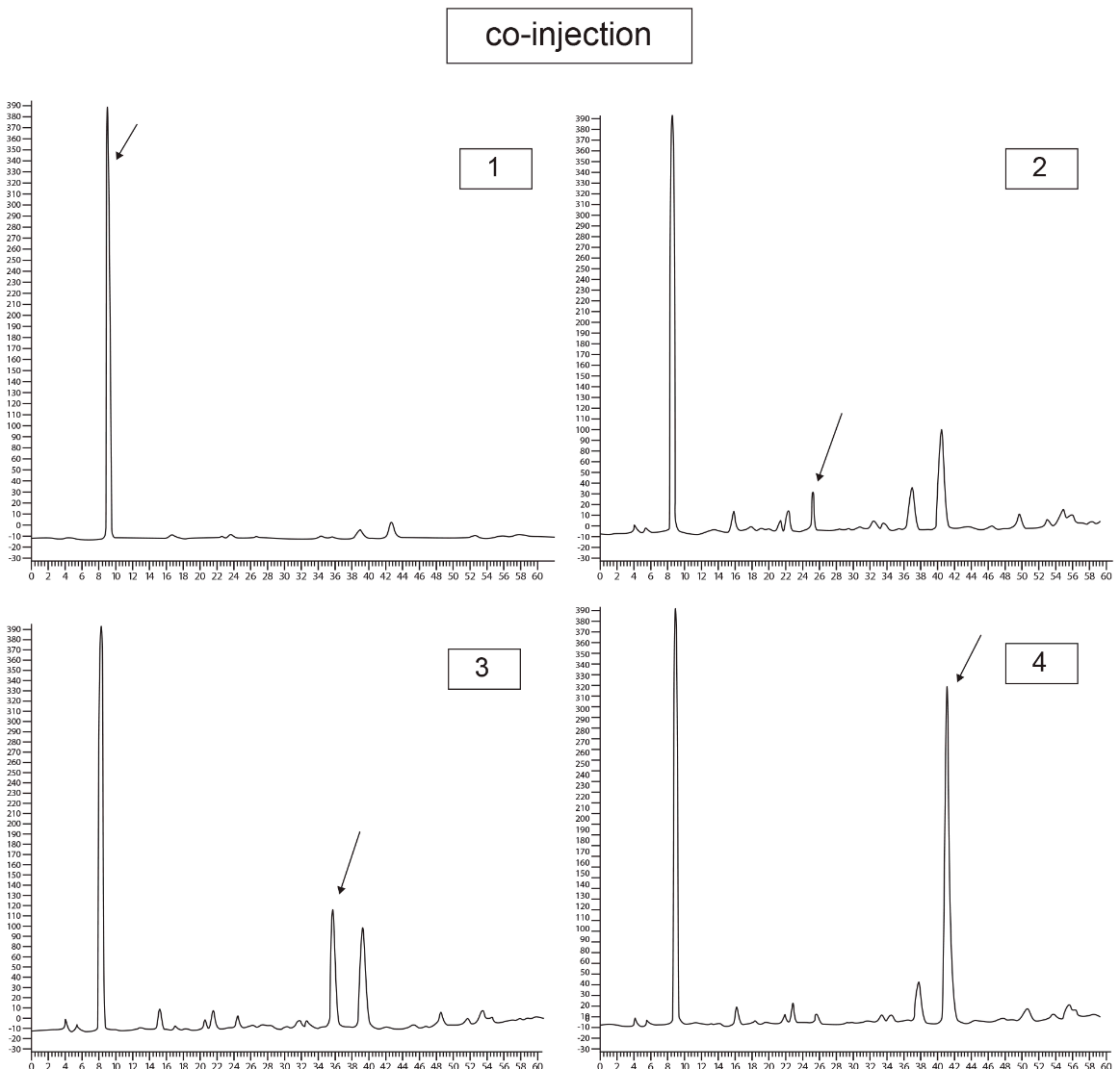
Chromatograms obtained by the coinjection of gallic acid (1); catechin (2); epicatechin (3) and epigallocatechin (4) standards with the EtOAc phase of acetone extract of *A. occidentale* barks.

The RT and areas of the peaks of interest resulting from coinjection of the standards and EtOAc phase of *A. occidentale* are shown in Table 2. The RT obtained for the standard substances were: 6.71 min for gallic acid, 19.93 min for catechin, 28.39 min for epicatechin and 30.53 for epigallocatechin. No new peaks or changes in the UV spectra were observed following coinjection of these substances. Furthermore, a significant increase in the area of the peaks attributed to the compounds was evident after coinjection, indicating higher concentrations and providing evidence of the presence of these phenols in the phase. The chromatograms of the standards and sample coinjection are shown in Figure 2.

These results are consistent with data reported in the literature, indicating a high content of phenolic compounds in the stem bark of *A. occidentale* [16]. Fujita [17] also reported the presence of flavonoids, catechins and tannins, as well as other compounds. The presence of tannins in the EtOAc phase of the stem bark of *A. occidentale* was previously demonstrated by Mota et al. [4].

### 2.2. Validation and Quantitation of Compounds

With regard to the peaks for the four phenolic compounds, all presented good resolution (Rs values ranging from 2.22 for epigallocatechin to 13.66 for epicatechin) and high N values (6839 for gallic acid to 45,105 for epicatechin), showing the method to be effective for separating the compounds [18]. In addition, T values ranged from 0.8 to 1.0, showing that the peaks are symmetric. Although the k’ values, denoting the rate of compound migration in the column, were considered satisfactory [19], gallic acid had the lowest value (0.79), indicating that this substance elutes rapidly, due to its higher polarity (epigallocatechin, the least polar compound, exhibited the highest value = 7.14). Concerning peak purity, values were close to 1.00, and hence the method can be considered selective for the compounds studied.

In addition, the chromatographic method proved to be precise, presenting correlation (RSD) below 5%, in accordance with the recommendations of Resolution 899 [20]. Furthermore, the average concentrations of gallic acid, catechin and epicatechin obtained on days 1 and 2 were compared using the *t*-test and were considered statistically similar (*p* > 0.05).

Accuracy is a parameter indicating the concordance of the results obtained by the method relative to the true value. The results obtained for gallic acid, catechin and epicatechin are given in Table 3. Slightly lower values were identified for gallic acid, indicating that the method may not be as reliable for exact quantification of this compound. For the catechins, however, values approaching 100% were observed, indicating a high recovery rate.

The analytical curves were linear over a wide concentration range and correlation coefficients were greater than 0.99. Table 4 shows the equations and concentrations of gallic acid, catechin and epicatechin in the EtOAc phase of the extract of the stem bark of *A. occidentale*.

Regarding the robustness of the method, no statistical difference was observed in comparisons of the concentrations measured by the method under nominal conditions of gallic acid when the oven temperature was increased to 35 °C (*p* < 0.05). Gallic acid, an ionizable substance, may have been affected by changes in column temperature. Other variations (oven at 25 °C and brand of acetonitrile solvent) produced no statistically significant differences. For catechin, there was no statistical difference (*p* > 0.05) between concentrations measured under nominal conditions and altered conditions.

The results indicated a high concentration of gallic acid in the phase. Previous studies have shown that gallic acid has a cardioprotective effect against cardiotoxicity induced by doxorubicin, attributed to the acid’s antioxidant property [21]. Furthermore, this compound exhibits cytotoxicity against cancer cells without damaging normal cells [22]. Gallic acid can be used to treat diabetes and albuminuria, and also displays a variety of pharmacological properties including antioxidant, antitumor, anti-inflammatory, antibacterial and antiviral [23,24,25,26,27,28,29,30,31].

Catechins also confer health benefits, attributed to their antioxidant activity, including anti-allergy, antimicrobial and anticancer effects [32] and aid in heart disease [33].

Several studies have suggested a correlation between the biological activities observed for flavonoids and use of the species containing these compounds in folk medicine. Nijveldt et al. [34] and Ondotuya et al. [35] demonstrated the antioxidant and anti-inflammatory properties of flavonoids. The presence of a phenolic ring and hydroxyl groups in the structure of these compounds may contribute to the activity of scavenging free radicals and inhibition of arachidonic acid synthesis [36].

Flavones and catechins seem to be more effective in protecting the body against reactive oxygen species, where epicatechin is one of the most powerful sequesters. The ability to inhibit degranulation of neutrophils and reduce complement activation were also shown in these studies.

Further studies should be conducted focusing on the safety and efficacy of the EtOAc phase, allowing its use in novel alternative therapies for inflammatory diseases.

### 2.3. Anti-Inflammatory Activity

#### 2.3.1. Effect of EtOAc Phases on Carrageenan, Bradykinin and Prostaglandin-induced Mice Paw Edema

**Figure 3:**
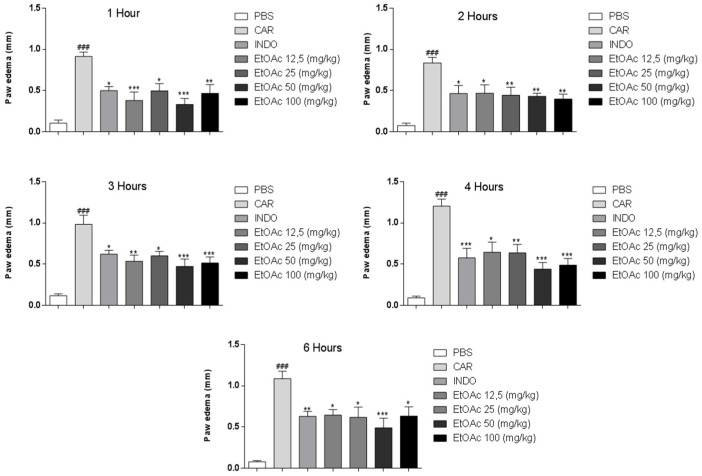
Reduction of paw edema (induced by carrageenan challenge) in the presence of EtOAc of *A. occidentale* at 1, 2, 3, 4 and 6 h. Results were expressed as mean SEM and analysed by graphpad Prism (version 5.0, Graph Pad Software, San Diego, CA, USA) using ANOVA followed by the test Dunnett or Mann–Whitney, where all groups were compared. ### *p* < 0.001 versus PBS group and * *p* < 0.05; ** *p* < 0.01; *** *p* < 0.001 versus carragenan group.

The results in Figure 3 show that all doses of the EtOAc phase reduced carrageenan-induced paw edema. A significant antiedema effect of the EtOAc phases was observed at 1 h (46%–64%), 2 h (44%–52%), 3 h (39%–52%), 4 h (47%–63%) and 6 h (41%–55%). Furthermore, indomethacin reduced carrageenan-induced edema at all time points.

The best-performing concentrations (50 and 100 mg/kg) significantly reduced paw edema (*p* < 0.05, *p* < 0.01 and *p* < 0.001). The optimal concentration was 50 mg/kg, proving effective for all time periods assessed, reducing edema by 49%–64% (*p* < 0.01 and *p* < 0.001).

**Figure 4:**
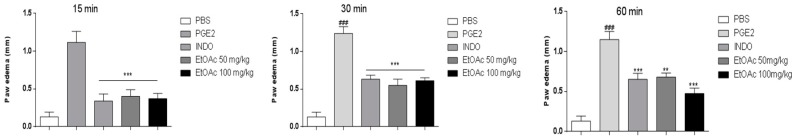
Reduction of paw edema (induced by the PGE challenge) in the presence of EtOAc of *A. occidentale* at 15, 30 and 60 min. Results were expressed as mean SEM and analysed by graphpad Prism using ANOVA followed by the test Dunnett or Mann–Whitney, where all groups were compared. ### *p* < 0.001 versus PBS group and ** *p* < 0.01; *** *p* < 0.001 versus carragenan group.

**Figure 5:**
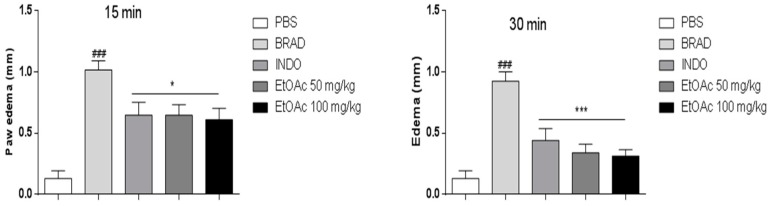
Reduction of paw edema (induced by bradykinin challenge) in the presence of EtOAc of *A. occidentale* at 15 and 30 min. Results were expressed as mean SEM and analysed by graphpad Prism using ANOVA followed by the test Dunnett or Mann–Whitney, where all groups were compared. ### *p* < 0.001 versus PBS group and * *p* < 0.05; *** *p* < 0.001 versus carragenan group.

A significant antiedema effect of EtOAc phase was observed at 15 (63% and 67%), 30 (56% and 51%) and 60 (41% and 59%) min after the PGE2 challenge (Figure 4) and at 15 (36% and 40%) and 30 (63% and 66%) min after the bradykinin challenge (Figure 5), at 50 and 100 mg/kg of the EtOAc phase, respectively.

Acute inflammation is characterized by coordinated activation of several signalling pathways that regulate the expression of mediators and cytokines with pro and anti-inflammatory functions. [37,38]. An inflammatory condition involves induction of edema and recruitment of cells, predominantly neutrophils, to the site of inflammation, driven by several mediators of different origins controlling its phases from onset to resolution [39,40,41]. These models induce inflammatory responses, including edema and neutrophil infiltration, and have been widely used for the quantification of specific cell types and inflammation-related soluble factors [39,42,43]. Recent studies have shown that carrageenan also induces the release of peripheral nitric acid sustained by TNF-α, Interferon–y (IFN-y) and IL-1β, which, in turn, induce an increase in levels of inducible nitric oxide synthase (INOS) in a host of different cells [44,45].

Carrageenan-induced paw edema is an acute inflammation model widely used for assessing antiinflammatory components [46]. The acute inflammatory response induced by the injection of carrageenan entails two phases involving the sequential release of various mediators [47,48]. The initial phase occurs within the first hour of exposure, triggering the release of histamine, serotonin, bradykinin and prostaglandins. After approximately 1 h, polymorphonuclear cells (mainly neutrophils) are recruited and continue the production of prostaglandin and nitric oxide [49].

The results of the present study indicate that all the EtOAc phase concentrations exerted a dose and time-dependent effect in paw edema reduction after the carrageenan challenge.

#### 2.3.2. Decrease in Carrageenan-Induced Peritonitis by the EtOAc Phase

According to Loram et al. [50], the peritonitis model produces inflammation in a slow and prolonged manner, allowing cell migration to be analyzed and quantified, as well as the involvement of cytokine, enzymes and chemical mediators. Peritonitis can be induced by a number of different phlogistic agents, including carrageenan. After observing the anti-inflammatory effects in the paw edema model, the most effective concentrations were selected for application in the peritonitis model to confirm the anti-inflammatory potential of the EtOAc phase of *A. occidentale*.

**Figure 6:**
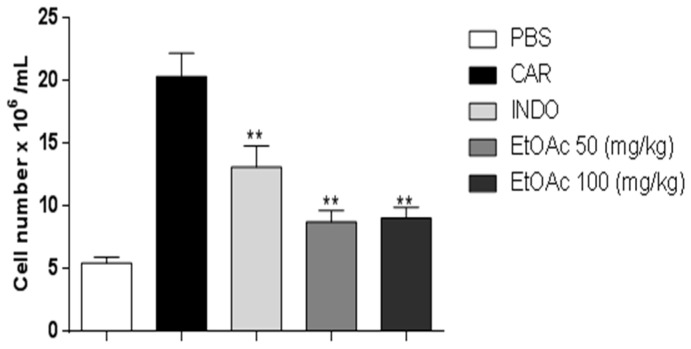
Reduction of total cell count in the presence of the EtOAc of *A. occidentale*. Statistical analysis performed using Student´s *t*-test. ** *p* < 0.01.

Peritonitis was measured 4 h after the carrageenan challenge. As shown in Figure 6, carrageenan challenge led to an increase in total cell numbers in the peritoneal cavity (at 4 h). In addition, administration of the EtOAc phase at doses of 50 mg/kg and 100 mg/kg inhibited carrageenan-induced leukocyte number in the peritoneal cavity at 4 h (57%). Moreover, indomethacin reduced leukocyte number induced by carrageenan for all time points.

#### 2.3.3. Effect of EtOAc Phases on Leukocyte Subsets in pPeritoneum

**Figure 7:**
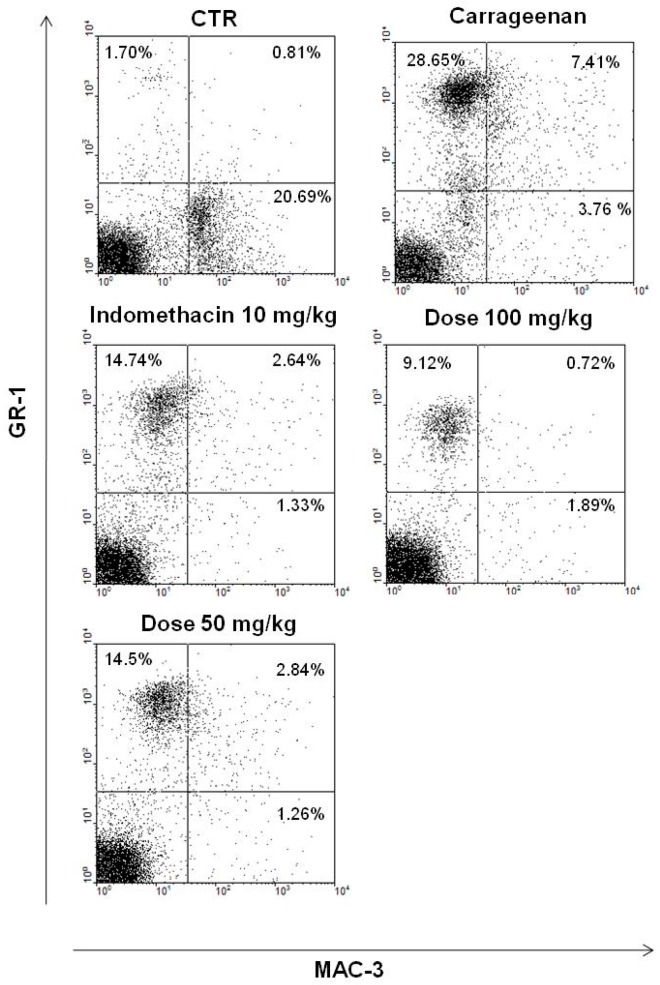
Reduction of differential neutrophil count, induced by carrageenan, in the presence of EtOAc of *A. occidentale*. Data was analyzed using the WinMDI 2.9 software (Joseph Trotter, Scripps Research Institute, San Diego, CA, USA).

Upon confirmation that the EtOAc phase reduced peritoneal exudate leukocyte number, flow cytometry was used to identify which cell subpopulations were affected. Four hours after challenge with carrageenan, a significant increase (28.6%) in neutrophils (Gr1+) at the site was observed. Moreover, the EtOAc phase (50 and 100 mg/kg) reduced the number of neutrophils to 14.5% and 9.1%, respectively, relative to the carrageenan group (Figure 7). In addition, indomethacin reduced the number of neutrophils to 14.74% (Figure 7). Furthermore, no alterations were observed in monocyte (Gr1+Mac3+) and macrophage (Mac3+) cell numbers 4 h after carrageenan injection or EtOAc treatment (Figure 7).

#### 2.3.4. Effect of EtOAc Phase on IL-1β, IL-6, IL-10 and TNF-α

The inflammation induced by carrageenan involves cellular migration, plasma exudation, and the production of mediators, such as nitric oxide, prostaglandin E2, interleukins IL-1β, IL-6, IL-10 and tumoral necrosis factor (TNF-α) [50]. These mediators are able to promote the recruitment of neutrophils in various experimental models. Evidence suggests these proinflammatory cytokines help propagate the extension of a local or systemic inflammatory process [51].

**Figure 8:**
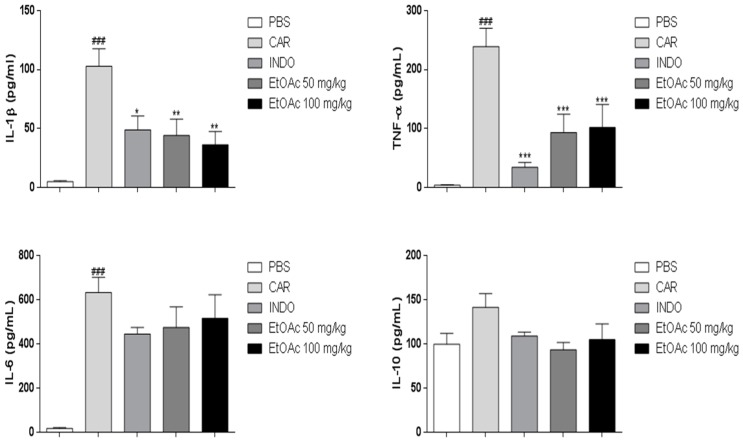
Levels of IL-1b, TNF-α, IL-6 and IL-10 (produced after carrageenan administration) in the presence of the ethyl acetate phase (EtOAc) of *A. occidentale*. Results were expressed as mean SEM and analysed by graphpad Prism using ANOVA followed by the test Dunnett or Mann–Whitney, where all groups were compared. ### *p* < 0.001 versus PBS group and * *p* < 0.05; ** *p* < 0.01; *** *p* < 0.001 versus the carragenan group.

The observed effect of EtOAc phase on the process of inhibiting carrageenan-induced leukocyte migration may be associated with the inhibition of synthesis of various mediators and cytokines involved in cellular migration. Figure 8 shows that carrageenan induced an increase in levels of TNF-α, IL-1β, IL-6 and IL-10 cytokines. EtOAc phase (50, 100 mg/kg) pretreatment significantly reduced TNF-α (61% and 58%) and IL-1β (57% and 65%) (Figure 8). However, EtOAc phases did not affect levels of IL-6 and IL-10 levels as compared to the carrageenan group. On the other hand, indomethacin promoted a significant reduction in TNF-α and IL-1β levels in peritoneal fluid.

Therefore, the inhibitory effect of the EtOAc phase against the influx of leukocytes in the peritoneal cavity of mice induced by carrageenan might be attributed to the suppression of the effect of proinflammatory cytokines such as TNF-α and IL-1β. These results corroborate findings of previous studies showing a possible anti-inflammatory effect of *A. occidentale* [4,5,15,52] and also elucidate mechanisms of action related to neutrophil migration and synthesis of TNF- α and IL-1β.

The Anacardiaceae family has broad use in folk medicine, particularly as an anti-inflammatory and antioxidant. [4,5,10]. Phytochemical analysis in different species of this family show the presence of constituents that have anti-inflammatory activity, such as gallic acid, catechin, epicatechin, ellagic acid [4,5,15,52,53,54,55,56,57].

Other families known for their popular use as anti-inflammatories were previously analyzed in order to correlate the biological effects of the presence of some phytochemicals. Soobrattee et al. [58] evaluated the antioxidant activity of various compounds, concluding that the highest efficacy was exhibited by gallic acid and rosmarinic acid, which were considered by the authors as good natural antioxidants, whose activity plays an important role in inflammatory processes.

Studies involving *Schinus terebinthifolius* have associated the presence of catechin and gallic acid with its popular use as an anti-inflammatory and antioxidant [59,60,61]. Bernardes et al. [62], using *S. terebinthifolius* extract, showed a reduction in the inflammatory process exacerbated in their studies and found antimycobacterial activity attributed to the flavonoids identified in this extract. Studies of the *Myracrodruon urundeuva*, also known in ethnopharmacology for its anti-inflammatory action, confirmed the presence of tannins and flavonoids in various parts of the plant [63,64].

Zocoler et al. [65] demonstrated a strong relationship between anti-inflammatory activity of the *Stryphnodedron obovatum* with the presence of gallic acid, epigallocatechin, and gallocatechin. Araújo-Neto et al. [66] have shown the anti-inflammatory effect of tannins and flavonoids in studies with *Sideroxylon obtusifolium* on paw edema induced by carrageenan and peritoneal leukocyte infiltrate in rats. Another study using a model of wounds and skin healing in rats showed good topical anti-inflammatory and healing activity of this species [67]. The anti-inflammatory mechanism of action of the *A. occidentale* EtOAc phase was evidenced in the present study and there is support in the literature that the phenolic compounds, whose presence has been demonstrated by the analytical method developed, play a role in inhibiting the inflammatory process [58,59,61,66].

Considering the levels of gallic acid, catechin and epicatechin found in the fraction assayed by HPLC (Table 4), the corresponding amounts in a 50 mg/kg dose (which demonstrated effectiveness) shall be 9.94; 0.34 and 0.58 µg/kg, respectively. These data can serve as the basis for future studies standardizing *A. occidentale* extract to ensure the same anti-inflammatory response.

## 3. Materials and Methods

### 3.1. Plant Material

Stem bark of *Anacardium occidentale* (cashew) was collected in April 2012, in the morning period, from plants located at the Center for Food Research and Processing (NUPPA) of the Federal University of Paraíba (UFPB) in João Pessoa (Paraíba State), Brazil, and identified at the Herbarium of the Department of Biology of the UFPB, where a voucher specimen is deposited in duplicate along with exsiccates of the botanical material, under registration number JPB 4.177. The material collected was dried at 40 °C in an oven with circulating air and milled in a cutting mill.

### 3.2. Extract Preparation

Acetone:water (70:30 *v*/*v*) extract was obtained by maceration of the bark powder with a plant:solvent ratio of 1:10 (*w*/*v*) for 72 h at room temperature with periodic shaking every 24 h. The resultant extract was submitted to liquid-liquid extraction with ethyl acetate. The aqueous phase was discarded and the EtOAc phase concentrated in a rotary evaporator under reduced pressure, producing a solid reddish-chestnut residue, following the method proposed by Haslam [68]. For the chromatographic analysis, the EtOAc phase was diluted in methanol: water 1:1 (*v*/*v*) to a concentration of 4 mg/mL.

### 3.3. Method Development

The method was performed on a Shimadzu System consisting of a chromatograph (Shimadzu, Tokyo, Japan), equipped with a LC-10AT pump, a UV/Diode Array Detector (SPD-M20A) at 280 nm, a DGU-20A5 degasser, a column oven (CTO-20A), and a SIL-20A autoinjector. The software Class Vp version 5.0 (Shimadzu, Tokyo, Japan) was used for data integration. The column was a C18 Phenomenex (250 mm × 4.60 mm; 5 μm particle diameter; granulometry of 100 Å) (Phenomenex, Torrance, CA, USA) maintained at 30 °C coupled to a C18 precolumn (Phenomenex, 5 µm particle diameter) (Phenomenex, Torrance, CA, USA).

HPLC grade acetonitrile and methanol were purchased from J. T. Baker^®^ (Phillisburg, NJ, USA) and Panreac^®^ (Barcelona, Spain). HPLC grade acetic acid was supplied by Vetec^®^ (Duque de Caxias, Rio de Janeiro, Brazil). Standards of phenolic compounds were obtained from Sigma-Aldrich (St. Louis, MO, USA) and Merck (Darmstadt, Germany). Water was purified using a Milli-Q system from Millipore (Milford, MA, USA). Mobile phase was filtered and degassed by passing it through 0.22 µm Nylon filters under vacuum.

Exploratory gradient starting with 5% Phase B (acetonitrile) and 95% of Phase A (0.3% acetic acid) until 100% Phase B were tested in order to establish satisfactory conditions for the chromatographic analysis of the EtOAc phase of *A. occidentale* (Table 1).

Stock standard solutions at 1 mg/mL for gallic acid and 0.5 mg/mL for the other standard compounds were prepared by dissolving each analytical standard in methanol. For preparation of the solutions for analysis, the samples were diluted with methanol:water (1:1).

Before injection, all solutions were filtered through a 0.45 µm Nylon membrane (injection volume = 20 µL).

### 3.4. Identification of Compounds

After establishing the analytical parameters, the method was applied to standards of phenolic compounds available: gallic acid, chlorogenic acid, catechin, epicatechin, rutin, luteolin, epigallocatechin, salicylic acid, gentisic acid, rosmarinic acid, 3,5-dihidroxybenzoic acid, 4-hydroxybenzoic acid, 3,4-dihydroxybenzoic acid, varying the wave length. The RT and UV absorption spectra obtained for each substance were compared to the chromatogram peaks of the sample. A coinjection run was then carried out with these standards (gallic acid, catechin, epicatechin and epigallocatechin) that had similar RT and UV spectra to the sample peaks and the *A. occidentale* EtOAc phase in order to detect the appearance of new peaks or changes in the area of the corresponding peak compared to the chromatogram of the phase alone. Thus, four solutions were prepared by mixing the EtOAc phase at 4 mg/mL for the solution of the corresponding standard substance at 0.5 mg/mL. A solution containing only the EtOAc phase at 2 mg/mL was prepared for the analysis, allowing comparison of the resulting peak areas with the chromatogram of the peaks from coinjection.

Parameters of system suitability related to the peaks such as number of theoretical plates (N), resolution (Rs), tailing (T), peak purity, capacity factor (k′) were evaluated according to the American Pharmacopoeia [18]. 

### 3.5. Validation

The method was validated according to RE 899 [3]. The parameters evaluated were linearity, accuracy, precision, specificity and robustness for gallic acid, catechin and epicatechin, with detection wave length set at 280 nm. Statistical analyses were performed using the software GraphPad Software Inc. (Version 5.0, Graph Pad Software, San Diego, CA, USA). A probability level of less than 5% (*p* < 0.05) was considered significant.

The specificity of the method was evaluated by the purity of each peak studied, using a UV/DAD detector. The ideal value is 1, indicating the absence of co-eluting substances. For linearity evaluation, the calibration curve contained six levels (from 100 to 600 µg/mL for gallic acid, 2 to 60 µg/mL for catechin and 5 to 100 µg/mL for epicatechin) and all analyses were carried out in triplicate. The evaluation of the intraday precision was performed by analyzing 6 samples at 2 mg/mL of the EtOAc phase of *A. occidentale*. The results were pooled and the Relative Standard Deviation (RSD) was calculated from the mean of 6 determinations of each analyte (intraday precision) and 12 determinations of each analyte (interday precision). In addition, Student’s *t-*test was performed to compare the average concentrations of each analyte on each of the days evaluated. Statistical significance was set at 5% (*p* < 0.05). Accuracy was tested using recovery studies performed in triplicate by spiking the standard at three different concentrations in the EtOAc phase solution at 2 mg/mL. Robustness of the method was checked by varying the column temperature (25, 30 and 35 °C) and the brand of the solvent used for phase B (J. T. Baker^®^ and Panreac^®^). The robustness of the method was evaluated by analysis of variance (ANOVA).

### 3.6. Anti-Inflammatory Activity

#### 3.6.1. Animals

Female Swiss albino mice (2 months old) were housed in a temperature-controlled room and received water and food *ad libitum.* After manipulation, euthanasia was employed by cervical dislocation. The study was performed according to the Guidelines on Ethical Standards for Investigation of Experimental Pain in Animals [69], after approval of protocol No. 0111/12 by the Institutional Ethics Committee of the Biotechnology Center (CBiotec, UFPB, João Pessoa, Brazil).

#### 3.6.2. Inflammatory Paw Edema

Mice were subdivided into groups (*n* = 6) treated with different doses of EtOAc phase at 12.5, 25, 50 and 100 mg/kg/weight (p.o.) one hour before intraplantar injections of phlogistic agents. To induce inflammation, mice received subcutaneous injections into the plantar surface of Carrageenan 2.5% (CAR), Bradykinin 6 nmol/paw (BRAD) and prostaglandin E2 5 µg/paw (PGE2) in 20 μL of phosphate buffered saline (PBS) in the right hind paw and 20 μL of PBS in the left hind paw. Indomethacin (10 mg/kg) was used as a control antiinflammatory and injected i.p. one hour before intraplantar injections. The resultant edema was measured using a digital micrometer and expressed as the difference in thickness (mm) between the challenged paw and saline-injected paw.

#### 3.6.3. Peritoneal Inflammation Model

Carrageenan-induced peritonitis was promoted as previously described in Montanher et al. [70] and Pagano et al. [71]. Carrageenan (500 µg/mL) was injected i.p. one hour after injection of doses of the EtOAc phase at concentrations of 50 and 100 mg/Kg. Indomethacin (5 mg/kg p.o.) was used as a positive control. Four hours after challenge, the animals were euthanized and peritoneal cavity lavage performed with 10 mL of cold PBS. Exudate was retrieved and the number of leukocytes present was determined by optical microscopy on a Hemocytometer using Turk’s solution (0.01% crystal violet in 3% acetic acid). Finally, the exudates were centrifuged and an aliquot of the supernatant was collected and stored at −20 °C for cytokine analysis.

#### 3.6.4. Measurement of Cytokine Levels by ELISA

TNF-α, IL-1β, IL-6 and IL-10 levels in the peritoneal fluid were evaluated by sandwich ELISA using kits specifically for mice, according to the manufacturer’s instructions (eBioScience, San Diego, CA, USA).

#### 3.6.5. Phenotyping of Leukocytes

Analysis of cell populations in the peritoneal cavity was performed by flow cytometry based on size and granularity, using anti-Gr-1 (eBioScience) to identify neutrophils and anti-Mac-3 (eBioScience, San Diego, CA, USA) to identify macrophages. Leukocytes collected from peritoneal lavage were stained with the antibody kits (eBioScience) according to the manufacturer’s protocol. Subsequently, 1 × 10**^−6^** cells/mL were incubated with a saturating amount of anti-Gr-1 PE conjugated and anti-Mac-3FITC conjugated for 30 min at 4 °C. Cells were then washed with cold PBS and resuspended in PBS. Analysis of Gr-1 and Mac-3 expression was performed using a FACSCaliburTM flow cytometer (San Diego, CA, USA).

For these experiments, data were acquired in a mode of 10,000 events. The data were analyzed by WinMDI software (Version 2.9, Joseph Trotter, Scripps Research Institute, San Diego, CA, USA).

### 3.7. Statistical Analysis

All data were expressed as mean ± SEM and treated statistically employing Student´s *t*-test, Analysis of Variance (ANOVA) was performed followed by the Dunnett or Mann–Whitney test and analyzed using the GraphPad Prism^®^ program version 5.0 (Graph Pad Software, San Diego, CA, USA).Values of *p* < 0.05, 0.01 and 0.001 were considered statistically significant results.

## 4. Conclusions

This study showed anti-inflammatory properties of oral therapy using EtOAc *Anacardium occidentale* phase in a murine model. These results are associated, at least in part, to the presence of the phytochemicals identified (gallic acid, catechin, epicatechin and epigallocatechin). The HPLC method developed proved suitable for the analysis of the *A. occidentale* EtOAc phase. It is simple, specific, accurate, precise and linear, does not involve laborious time-consuming sample preparation and can be considered suitable for the routine quantitative analysis of gallic acid, catechin, and epicatechin in the EtOAc phase of *A. occidentale.*

In addition, the phase was found to exert an effect during the early and acute stages of inflammation induced by different phlogistic agents. Anti-edematogenic activity was observed, associated with the inhibition of mediators such as PGE and Bradykinin, inhibition of cell migration to the site of inflammation, and its interference in levels of proinflammatory cytokines (TNF-α and IL-1β). These results suggest that the EtOAc phase tested plays a modulatory role in the inflammatory response.

## Figures and Tables

**Table 1:** Gradient elution tested in development of the HPLC chromatographic method for analysis of EtOAc phase of *A. occidentale*.

Run Number	Gradient Elution	
	Time Interval	Proportion of Phase B
1	0–60 min	5%–100%
2	0–60 min	5%–50%
3	0–60 min	5%–30%
4	0–25 min 25.01–60 min	5%–15% 15%–20%
5	0–14 min 14.01–30 min 30.01–60 min	7%–12% 12%–12% 12%–25%
6	0–10 min 10.01–30 min	7%–11% 11%–11%
7 *	0–18 min 18.01–35 min 36.00–45 min	7%–12% 12%–12% 7%–7%

* Gradient elution established for subsequent analysis and method validation.

**Table 2:** Peak areas of interest on chromatogram for *A. occidentale* EtOAc fraction with and without coinjection of gallic acid, catechin, epicatechin and epigallocatechin.

Result of Analysis with EtOAc Phase of *A. occidentale*		Result of Coinjection Analysis		
Retention Time (min)	Area (mAU.s)	Coinjection with	Retention Time (min)	Area (mAU.s)
6.79	19,837.2	Gallic acid	6.72	26,015.2
19.83	167.1	Catechin	19.48	540.8
28.44	1447	Epicatechin	28.36	3293
30.96	2918	Epigallocatechin	31.8	11,233.5

**Table 3:** Accuracy of HPLC method for gallic acid, catechin and epicatechin at three concentration levels.

Compound	Concentration Level (μg/mL)	Expected Concentration (μg/mL)	Recovered Concentration (μg/mL)	Recovery Percentage (%)	RSD * (%)
	100	482.90	426.22	88.26	1.27
Gallic acid	200	582.90	501.78	86.10	2.98
	300	682.91	492.59	72.13	1.74
	10	22.80	21.47	94.17	1.85
Catechin	20	32.79	32.51	99.13	0.99
	40	52.80	50.58	95.79	4.94
	25	46.19	47.14	102.06	1.35
Epicatechin	50	71.19	71.86	100.95	2.02
	75	96.19	96.02	99.82	4.52

* RSD = Relative Standard Desviation.

**Table 4:** Quantitation of gallic acid, catechin and epicatechin in EtOAc phase of extract of stem bark of *A. occidentale.*

Suggestive Peak	Equation of Line	Peak Area (mAU.s)	Quantitation (μg/mL)	Concentration in Extract (μg/g)
Gallic acid	y = 57,270x + 565,300 (R^2^ = 0.9968; RSD = 3%)	23,336,667	397.61	198.81
Catechin	y = 16,770x − 8922 (R^2^ = 0.9960; RSD = 4.9%)	222,170.2	13.78	6.89
Epicatechin	y = 16,780x − 22,620 (R^2^ = 0.9935; RSD = 5%)	367,368.8	23.24	11.62
